# Presence of Avian Influenza Viruses in Waterfowl and Wetlands during Summer 2010 in California: Are Resident Birds a Potential Reservoir?

**DOI:** 10.1371/journal.pone.0031471

**Published:** 2012-02-06

**Authors:** Viviane Hénaux, Michael D. Samuel, Robert J. Dusek, Joseph P. Fleskes, Hon S. Ip

**Affiliations:** 1 Department of Forest and Wildlife Ecology, University of Wisconsin, Madison, Wisconsin, United States of America; 2 Wisconsin Cooperative Wildlife Research Unit, U.S. Geological Survey, Madison, Wisconsin, United States of America; 3 National Wildlife Health Center, U.S. Geological Survey, Madison, Wisconsin, United States of America; 4 Western Ecological Research Center, U.S. Geological Survey, Dixon, California, United States of America; Erasmus Medical Center, The Netherlands

## Abstract

Although wild waterfowl are the main reservoir for low pathogenic avian influenza viruses (LPAIv), the environment plays a critical role for the circulation and persistence of AIv. LPAIv may persist for extended periods in cold environments, suggesting that waterfowl breeding areas in the northern hemisphere may be an important reservoir for AIv in contrast to the warmer southern wintering areas. We evaluated whether southern wetlands, with relatively small populations (thousands) of resident waterfowl, maintain AIv in the summer, prior to the arrival of millions of migratory birds. We collected water and fecal samples at ten wetlands in two regions (Yolo Bypass and Sacramento Valley) of the California Central Valley during three bi-weekly intervals beginning in late July, 2010. We detected AIv in 29/367 fecal samples (7.9%) and 12/597 water samples (2.0%) by matrix real time Reverse Transcription Polymerase Chain Reaction (rRT-PCR). We isolated two H3N8, two H2N3, and one H4N8 among rRT-PCR positive fecal samples but no live virus from water samples. Detection of AIv RNA in fecal samples was higher from wetlands in the Sacramento Valley (11.9%) than in the Yolo Bypass (0.0%), but no difference was found for water samples (2.7 vs. 1.7%, respectively). Our study showed that low densities of hosts and unfavorable environmental conditions did not prevent LPAIv circulation during summer in California wetlands. Our findings justify further investigations to understand AIv dynamics in resident waterfowl populations, compare AIv subtypes between migratory and resident waterfowl, and assess the importance of local AIv as a source of infection for migratory birds.

## Introduction

Wild birds (orders Anseriformes and Charadriiformes) are capable of maintaining and spreading most subtypes of low pathogenic avian influenza viruses (LPAIv) [1]. LPAIv replicate primarily in the intestinal tract of infected birds, with large amounts of virus shed through feces into the environment [2]. Based on experimental studies, Hénaux and Samuel [3] estimated that virus excreted during the infectious period represented about 1,500 times the median bird infectious dose (BID_50_) for LPAIv. This level of contamination implies that the environment is critical to AIv transmission through the fecal/oral route [4]. Accordingly, recent modeling of LPAIv dynamics in wild waterfowl suggested that disease cannot be maintained in many populations without environmental transmission [5]–[6].

The role of the environment as a reservoir for AIv is also supported by the ability of LPAIv to persist in water for extended periods [7]–[9]. Experimental studies demonstrated that temperature greatly influences viral persistence, with an exponential decay of viral infectivity as temperature increases [7]. In addition, AIv are most stable in freshwater (i.e., low salinity) with pH between 7.4 and 8.2 [8], [10]–[11]. Prolonged infectivity in cold freshwater (≤4°C [2], [7], [9]) suggests that in the northern hemisphere (implied hereafter) AIv may persist longer in northern than southern waterfowl habitats, and infect migratory birds returning to breeding areas during spring [12]–[13]. In contrast, decreased survival in warmer water implies limited LPAIv persistence and transmission among non-migratory waterfowl during summer on southern wetland areas [7].

**Figure 1:**
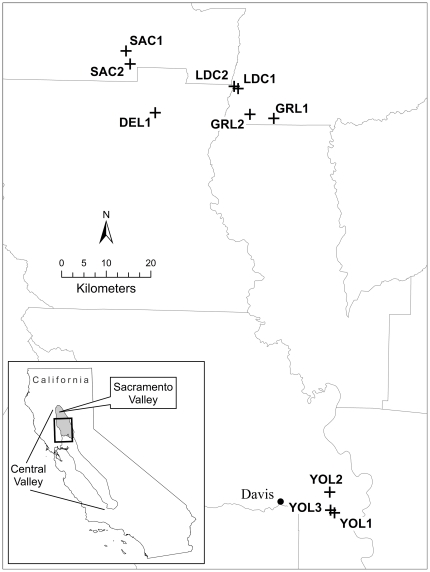
Location of the study wetlands. Water and fecal samples were collected during three periods across summer at all wetlands.

Although the transmission of AIv was documented in resident waterfowl in southern areas during winter [14], the role of local populations in the maintenance of AIv during summer is still unknown. Identifying the sources of AIv affecting wintering waterfowl (i.e., AIv circulating in migratory populations vs. present locally in the environment) would improve our understanding of the role of southern wetlands as a reservoir for AIv and migratory birds as AIv carriers, and help determine the risks related to the spread of AIv. The objective of our research was to evaluate the role of summer wetlands and resident waterfowl in California as potential reservoirs for AIv. We hypothesized that AIv subtypes would be unlikely to persist in these wetlands during the summer because of unfavorable environmental conditions (especially high temperatures) and absence of a sufficient waterfowl population to serve as an effective AIv reservoir. We collected up to 20 fecal samples from resident waterfowl and 20 water samples at ten wetlands in two regions of the California Central Valley (Figure 1) at bi-weekly intervals from late July to late August 2010; three wetlands were in the Yolo Bypass east of Davis, CA, and the other seven were 80–100 km north in the Sacramento Valley.

## Results

We detected AIv in 29/367 fecal samples (7.9±1.4% (SE)), and 12/597 water samples (2.0±0.6%) by AIv matrix gene real time Reverse Transcription-Polymerase Chain Reaction (rRT-PCR); three water samples leaked during shipping and could not be analyzed (Table 1). The proportion of AIv-positive samples was significantly higher (z = 3.62, *P*<0.001) in fecal than water samples. We found mean threshold cycle (Ct) values of 38.9±0.2 in water samples and 34.6±0.7 in fecal samples (t = −4.05, *P*<0.001) indicating higher virus concentrations in feces. Our probability of detecting AIv in fecal samples was higher on SACV than YOLO wetlands (11.9±2.1 vs. 0.0±1.5% [95%-CI: 0.0–2.9], respectively; z = 4.02, *P*<0.001), but there was no difference for water samples (2.7±0.8 vs. 1.7±1.0%, respectively; z = 0.758, *P* = 0.45). There was no significant difference in the detection rate among the three periods in fecal samples at SACV (5.0±4.9%, 14.9±3.3%, and 10.1±2.9%, respectively; χ^2^ = 2.2, 2 df, *P* = 0.33). Similarly, AIv detection in water samples at both SACV and YOLO was similar among the three periods (3.5±1.3%, 2.0±1.0%, and 0.05±0.05%, respectively; χ^2^ = 4.6, 2 df, *P* = 0.10). Of the rRT-PCR positive fecal samples, five were positive on virus isolation, resulting in a recovery rate of 17.2%. Ct values for these samples ranged from 30.6 to 38.4. Isolated viruses included two H3N8 (SAC1, late July; LDC2, mid-August), two H2N3 and one H4N8 (LDC1, mid-August). No virus was isolated from water samples.

**Table 1:** Number of AIv RNA-positive water and fecal samples at each wetland of the California Central Valley for the three sampling periods.

Wetland	Date	No. positive^a^/total water samples	No. positive^a^/total fecal samples
SAC1	Late July	**1/20**	**1/20** b
	Mid August	**1/20**	**3/20**
	Late August	0/20	**1/20** c
SAC2	Late July	0/20	-
	Mid August	**1/20**	**2/20**
	Late August	0/20	**5/20**
DEL1	Late July	0/20	-
	Mid August	0/20	0/1
	Late August	0/20	**3/20**
LDC1	Late July	0/20	-
	Mid August	**1/20** c	**5/20** b c
	Late August	0/20^c^	**1/20** c
LDC2	Late July	0/20	
	Mid August	0/19	**3/20** b
	Late August	0/20^c^	-
GRL1	Late July	**3/20**	-
	Mid August	0/20	0/13
	Late August	0/20	0/8
GRL2	Late July	**1/20**	-
	Mid August	0/20	**4/20** c
	Late August	**1/20**	**1/20**
YOL1	Late July^d^	**1/20**	0/5
	Mid August	0/20	0/20
	Late August	0/20	0/20
YOL2	Late July	**1/20**	0/20
	Mid August	0/18	0/20
	Late August	0/20	0/20
YOL3	Late July	0/20	-
	Mid August	**1/20**	-
	Late August	0/20^c^	0/20^c^

a For rRT-PCR result interpretation, we considered samples with a Ct value≤40.0 as AIv-positive.

b AIv isolates include one H3N8 at SAC1 in late July, one H4N8 and two H2N3 at LDC1 in mid-August, and one H3N8 at LDC2 in mid-August in fecal samples.

c Samples collected at a different wetland to that sampled previously.

d Presence of cows at this wetland at sampling time.

Mallards *Anas platyrhynchos* were the most abundant waterfowl species at study wetlands followed by cinnamon teal *A. cyanoptera*, gadwall *A. strepera*, ruddy duck *Oxyura jamaicensis*, and wood duck *Aix sponsa*. In late August, northern pintail *Anas acuta*, and northern shoveler *A. clypeata* were also observed at YOL1.

Mean site-specific water temperatures ranged from 16.9 to 30.6°C, pH from 7.0 to 10.0, conductivity from 113.1 to 1246.8 µS/cm, dissolved oxygen (DO) from 28.7 to 296.1 mg/L, turbidity from −2.6 to 873.3 NTU (Nephelometric Turbidity Units), and coliform concentration from 2 to 1600 MPN (Most Probable Number) over the course of the study.

## Discussion

Although waterfowl species are known to contribute to the dispersal of AIv from breeding to wintering areas [1], our study is the first to investigate the presence of virus in wetlands and resident waterfowl populations in southern wetlands during summer. We detected AIv RNA in 7.9% of fecal samples of resident waterfowl, with a higher detection probability in SACV than YOLO wetlands. The probability of detection of AIv RNA was significantly lower in water (2.0%) compared with feces. We isolated multiple influenza viruses (H3N8, H2N3, H4N8) from fecal samples at several SACV wetlands, indicating circulating LPAIv infections in resident duck species late into the summer.

We found a low detection of AIv RNA in water samples, although virus isolation in feces indicates ducks were shedding live virus into wetlands. However, virus dilution in wetlands is expected to reduce virus concentration and detection probability, as indicated by the higher RT-PCR Ct values in water. In laboratory experiments, LPAIv persist in water conditions similar to those measured during our study from a few days to a few months [7]–[8], [10], but there is limited information on the influence of natural wetland characteristics on virus persistence. Microorganisms and filter-feeding bivalves can reduce AIv survival and infectivity [15]–[16]. Although we conducted detailed statistical analyses, we were not able to show any significant influence of water characteristics, concentrations of coliform bacteria, or bird abundance on AIv detection (all *P*>0.05; results not shown). We suspect the low detection rate and the limited range of conditions in our study affected this analysis. Our findings indicate the need for improved detection of AIv in water samples as well as the investigation of biotic and abiotic components affecting virus survival in natural environments.

We isolated several AIv subtypes from fecal samples indicating current infections of resident waterfowl and environmental contamination in California wetlands during summer. We obtained virus from 17.2% of rRT-PCR positive fecal samples which corresponds with the range reported in other studies (3 to 45% [17]–[20]). However, we did not isolate AIv from positive water samples. Variations in isolation rates among studies likely result from differences in sample methods (cloacal or oropharyngeal swab only, field- or lab-combined swabs, environmental samples), host species, and environmental characteristics. Although we sampled fresh feces the survival of AIv may be affected by temperature and humidity, with a loss of infectivity of HPAIv (H5N1) within 1 day at 25°C in dried feces [21]. Low viral titer (as observed at the end of the infectious period [3]), inactivated or non-infectious virus, and the presence of inhibitors [20] may also contribute to the low isolation rates. In water, UV radiation might inactivate virus in the water column and virus dilution may reduce isolation rates.

The reasons for higher detection of AIv RNA in feces at SACV vs. YOLO wetlands are unclear and may include a lower resident duck density at YOLO [22] or different proportions of naïve juvenile birds among these two regions. In summer 2008, LPAIv infection prevalence (by rRT-PCR) in live ducks was 9.1% (4/44) at Mendota Wildlife Area in the southern Central Valley (i.e., San Joaquin Valley), but only 1.1% at Lower Klamath NWR about 200 km north of the Central Valley [23]. Given that waterfowl are the primary source of environmental AIv, monitoring the distribution, species and densities of resident waterfowl several weeks prior to sampling, in relation to wetland habitat (e.g., presence of cows at YOL1 in late July) and management (e.g., water level), may help understand spatial heterogeneities in AIv distribution.

Our findings indicate that resident waterfowl populations in southern wetlands may serve as a source of virus for migratory ducks during winter. The prevalence of AIv infection in waterfowl wintering in the SACV and YOLO regions may reach up to 5% in some species [24]–[25] and further research is needed to evaluate the extent in which AIv circulating during summer can cause infection during winter. Among the AIv found in our study, H3N8 is commonly found in the Pacific and Central flyways [12], [25]–[31], and has been frequently detected in California. In contrast, H2N3 and H4N8 have been isolated from free-living aquatic birds in Alaska, Canada, and Texas [12], [29]–[31], but have not been previously reported in California. Comparing the genetic sequences of the AIv from our study with reference sequences may provide insight on the origin of these viruses and clarify the importance of summer virus persistence in LPAIv dynamics.

We sampled semi-permanent/permanent wetlands in July-August to minimize potential for virus from northern-breeding migrants. Adult male northern pintails are one of the first species to migrate into the Central Valley, arriving as early as the first week of August [32]. However, pintail abundance in our study area during early August was low (100s) and these early-arriving migrants concentrate on seasonal wetlands with high carbohydrate foods (i.e., seeds) needed to replenish reserves depleted by migration. At the semi-permanent/permanent wetlands we sampled, only local breeding populations (i.e., mallard, gadwall, cinnamon teal *Anas cyanoptera*, wood duck, american coot *Fulica americana*, pied-bill grebe *Podilymbus podiceps*, ibis *Plegadis chihi*, egret *Ardea alba*) were observed in late July-early August. Although migrants had increased to the thousands by our third sampling period, we observed migrant species on only two of the wetlands sampled at YOLO (i.e., several pintails and northern shovelers on YOL1 and several hundred shorebirds on YOL2), but did not detect AIv at these sites. These observations and the fact that AIv detection rate did not increase in August indicate that the limited number of early migrants did not likely contribute to the AIv pool.

Our findings suggest that cold environmental water temperature and high bird numbers may not be required to maintain AIv circulation. Although the low densities of resident waterfowl populations and unfavorable environmental conditions may impact virus circulation and epizootic dynamics (i.e. reduce transmission, decrease virus diversity), our findings showed that California waterfowl and wetlands may serve as a reservoir for AIv. Our findings justify further longer-term investigations about the dynamics of AIv infection in resident waterfowl populations to determine the importance of southern summer waterfowl areas as a potential source of infection for migratory wintering ducks, and to evaluate the potential to enhance virus exchange and favor virus reassortment through mixed infections [25]. Such information is basic for the understanding of AIv epidemiology and ecology.

## Methods

### Study areas and sample collection

About 10–15% of the wetlands in the Central Valley are semi-permanent or permanent and maintain summer water for the approximately 400,000 resident waterfowl (about 70% mallard *Anas Platyrhynchos*) that breed there ([22], California Dept. of Fish and Game, unpublished data). Most seasonal and semi-permanent wetlands in the Central Valley are managed primarily to provide food and refuge for wintering waterfowl. Managers schedule flooding and periodic disking or burning to encourage growth of swamp timothy, watergrass (*Echinochloa crusgalli*), and smartweed (*Polygonum*), or a mix of these and other wetland (e.g., alkali bulrush, *Juncus*, *Paspalum distichum*) or moist-soil plants [33]. These and the fall-flooded seasonal wetlands support several million migratory waterfowl during winter [22], [34].

To limit the uncertainty inherent to disease surveillance surveys and enhance detection probabilities [35], we conducted repeated sampling in time and space [36]. We monitored ten wetlands for AIv at five major waterfowl wintering areas in the California Central Valley. All wetlands studied were either on federal or state lands and permission for sampling was obtained from the manager of each area. This study did not involve endangered or protected species and no other specific permits were required. We sampled wetlands that have permanent water, are frequently used by resident waterfowl populations, and have historically hosted high densities of migratory waterfowl during winter. In the Sacramento Valley (SACV; 39.37°N, -121.97°E) we sampled two wetlands at the Sacramento National Wildlife Refuge (SAC1 and SAC2), one wetland at Delevan National Wildlife Refuge (DEL1), two wetlands at Little Dry Creek (LDC1 and LDC2), and two wetlands in Gray Lodge Wildlife Area (GRL1 and GRL2). SAC, DEL, GRL, and LDC wetlands were ≤30 km apart. In the Yolo Bypass (YOLO; 38.54°N, -121.61°E), we sampled three wetlands at Yolo Wildlife Area (YOL1, YOL2 and YOL3; Figure 1). Sampled wetlands were bordered by idle grasslands and located in a rice dominant agricultural landscape [37]. Wetlands were sampled at bi-weekly intervals from late July to late August 2010 (3 sampling periods). However, three wetlands were unexpectedly drained before the end of our sampling: LDC1 after the late July sampling, and LDC2 and YOL3 after the mid- August sampling. In these cases, sampling during the remaining period(s) occurred in an adjacent wetland (500–2300 m-distant). Size of wetlands sampled averaged 18 ha (SD = 16 ha) and ranged from 6–58 ha. There was no detectable water flow in any of the wetlands during the sampling period.

During each wetland sampling period, we collected 20 samples of 45 ml of surface water at representative wetland vegetation sites distributed throughout the wetland, within areas accessible by foot (≤1.2 m depth). At ten sample locations (every other water sample) we also measured water characteristics (temperature, pH, turbidity, dissolved oxygen (DO), and salinity) using a YSI 6920 V2-1 sonde (YSI Inc., Yellow Springs, OH). At YOL2 in late August these measurements were carried out only at three sample locations because water at other sample locations was too shallow (<15 cm depth). Approximate bird numbers, including primarily Anseriformes, and in a lower extent Ciconiiformes, Charadriiformes, Gruiformes, Pelecaniformes, and Podicipediformes, were recorded as low (<50 birds), moderate (50–100 birds), and high (>100 birds) based on binocular observations of open water areas for each wetland sampling period. Bird abundance at sampling time was an indicator of potential viral shedding so we could evaluate the probability of detecting AIv in wetlands with higher relative duck abundance. In large wetlands, we primarily sampled water areas used by ducks to increase AIv detection. During the first and third sampling periods, a composite sample of surface water consisting of four sub-samples from each wetland was collected and sent to a microbiology laboratory (Basic Lab., Chico, CA) to determine the concentration of coliform bacteria.

During each wetland sampling period we collected up to 20 fecal samples at ≥one waterfowl roost site (loafing or feeding location) along the wetland edge or on islands. Collection of fresh feces offers the opportunity to obtain information on the presence of AIv in wild bird populations without capturing birds [38]. At each site, we collected one sterile Dacron® swab sample per distinct fresh feces; although we did not collect samples from adjacent feces, we cannot exclude the possibility that some fecal samples collected in the same roost site were from the same individual. Because of the absence of fresh feces at GRL2 (mid August) and SAC1 (late August) we collected fecal samples at an adjacent wetland (300–2000 m-distant). Fresh fecal swabs were immediately placed into a 1.5 ml vial of viral transport media [39]. Fecal and water samples were kept cool in the field and shipped with blue ice within 24 hours of collection to the U. S. Geological Survey National Wildlife Health Center, Madison, WI, for laboratory analyses.

### Laboratory analyses

Molecular detection of AIv matrix was performed on all individual samples. RNA was extracted from 50 µl of the water or fecal swab sample and the presence of AIv tested according to the AIv matrix gene rRT-PCR method as described by Spackman et al. [40]. Results from rRT-PCR are reported in threshold cycle (Ct) values, which correspond to the number of rRT-PCR cycles required to detect nucleic acid (on a log_10_ scale); lower Ct levels indicate greater concentration of virus RNA in the sample. There is no recommended Ct limit value for the use of the Spackman et al. [40] method on environmental samples in the literature. However, data from the 2009 surveillance across the United States showed a linear decrease in virus isolation rate with increasing rRT-PCR Ct values in swab samples, with 20% recovery at Ct = 40.0 (n = 1624 samples, R^2^ = 0.99; Ip unpublished data). Individual samples with Ct-values≤40.0 were considered as positive and were further analyzed using the H5 and H7 rRT-PCR tests [40]–[41]. All matrix gene rRT-PCR test positive specimens were then tested by virus isolation in embryonating eggs [42]. Note that, in our study, no virus was isolated from samples with rRT-PCR Ct values >40.0, indicating no false negative test results related to the Ct cut-off value. Allantoic fluid from each egg was tested for the presence of hemagglutinating viruses using chicken and turkey red blood cells. Hemagglutination-negative samples were passaged at least once more and re-tested before the original samples were considered negative. Hemagglutination-positive samples were retested by rRT-PCR to identify AIv isolates. Virus subtyping (for all HA and NA subtypes) was conducted on positive samples from virus isolation by RT-PCR and sequence analysis as described by Hoffman et al. [43].
